# Combining Physiology and Transcriptome to Reveal Mechanisms of *Hosta* ‘Golden Cadet’ in Response to Alkali Stress

**DOI:** 10.3390/plants14040593

**Published:** 2025-02-15

**Authors:** Xiaogang Sun, Chunyao Zhu, Baizhou Li, Wei Ning, Jiahui Yin

**Affiliations:** Jilin Provincial Key Laboratory of Tree and Grass Genetics and Breeding, College of Forestry and Grassland Science, Jilin Agricultural University, Changchun 130118, China; sunxiaogang@jlau.edu.cn (X.S.); zcy6366@outlook.com (C.Z.); 13596447373@163.com (B.L.); ningwei@jlau.edu.cn (W.N.)

**Keywords:** alkali, *Hosta* ‘Golden Cadet’, physiological, transcript, response mechanism

## Abstract

As an ornamentally and medicinally worthy plant, *Hosta plantaginea* (Lam.) Aschers. has the adapted capacity to survive cold temperate monsoon climates in Northeastern China. However, its use is limited by the soil alkalization of urban gardens. Our pre-experiment found that *Hosta* ‘Golden Cadet’ has the potential to be alkali-tolerant. Hence, tissue-cultured seedlings of *Hosta* ‘Golden Cadet’ were used as experimental material. Its related growth, physiology, and transcripts were examined to reveal the molecular mechanism of *Hosta plantaginea* in response to alkali stress. The results show that the development of *Hosta* ‘Golden Cadet’ was affected by alkali stress. In comparison with the control, malondialdehyde (MDA) content increased by 4.28-fold at the 24th hour, superoxide dismutase (SOD) activity increased by 49% at the 6th hour, and peroxidase (POD) activity and soluble sugar (SS) content increased by 67% and 30% at the 12th hour, respectively. The RNA-seq analysis revealed that *Hosta* ‘Golden Cadet’ gene expressions at 0 h, 6 h, 12 h, 21 h and 48 h differed after 200 mmol/L NaHCO_3_ treatment. During 48 h under alkali stress, 2366 differentially expressed genes were found. The transcription factors MYB, AP2/ERF, and WRKY were activated in differentially expressed genes. The KEGG analysis found that phytohormone signaling pathways, starch and sucrose metabolism, and phenylpropane production were activated in *Hosta* ‘Golden Cadet’ in response to alkali stress. In summary, *Hosta* ‘Golden Cadet’ can reduce membrane damage by improving osmoregulation and antioxidant capacity, increase sucrose and starch metabolism, and regulate phenylpropane biosynthesis by activating transcription factors and inducing related phytohormone signaling, mitigating the effects of alkali toxicity. These findings guide an investigation into the mechanism of alkali tolerance in *Hosta* plants, screening alkali tolerance genes, and selecting and breeding novel alkali-tolerant *Hosta plantaginea* cultivars.

## 1. Introduction

As urbanization grows, water loss and alkalization in urban garden soils become more significant [1]. Salinization is a major abiotic stressor in urban garden environments, limiting plant growth, development, and survival [2]. Ornamental plants grow directly in urban soils and are influenced by soil salinity and available irrigation water during their development [3]. Jilin Province in China experiences a moderate monsoon climate with hot, rainy summers and cold, dry winters, resulting in a scarcity of decorative plant material [4]. Moreover, as a result of the region’s harsh meteorological circumstances and human activities, such as freeze–thaw cycles and intense evaporation, the soil of urban gardens is increasingly alkalized, with NaHCO_3_ and Na_2_CO_3_ dominating their composition [5,6]. Furthermore, groundwater is the primary source of irrigation water in this area and, with urbanization accelerating, is becoming increasingly polluted, with high levels of Ca^2+^ and Na^+^ and ions such as HCO_3_^−^, SO_4_^2−^, and Cl^−^, with HCO_3_^−^ being the most prevalent ion in groundwater [7]. This inhibits normal plant growth in the region. Alkali stress causes the same stress characteristics as salt stress, including osmotic stress, oxidative response, and ionic damage in the plant body, as well as an increase in reactive oxygen species (ROS), which damages intracellular components [8,9]. However, in combination with higher pH values (pH > 8.5), alkaline salts have much more damaging effects on plants than neutral salts [10,11]. Adaptation strategies to salt stress in ornamental garden plants are aimed at mitigating the effects of osmotic, ionic, and oxidative stress, as well as salinity-induced nutrient imbalances [12]. For example, *Echinacea* exhibited higher Na^+^ exclusion capacity and enhanced antioxidant activities of APX and SOD under salt stress [13]. *Eugenia myrtifolia* L. APX content and activity reduced saline conditions, although SOD content and activity in leaves rose [14]. In conclusion, ornamental plants that are adapted to their climatic circumstances and resistant to alkaline salts containing HCO_3_^−^ should be used in the landscaping of this region. Currently, there are limited investigations on the mechanism of alkali tolerance in garden plants [15,16]. As a result, understanding the mechanisms of alkali tolerance in garden plants is critical to improving and applying urban gardens in northeast China.

With its rapid development, transcriptome sequencing technology has been widely used in the study of plant response to saline and alkaline stress, as well as other types of abiotic stress, and a large number of genes involved in metabolism, photosynthesis, signaling, and the osmotic regulation of salinity-tolerant responses have been discovered [17]. Transcriptome sequencing was employed to show the mechanism of wild jujube’s alkaline tolerance response through a comprehensive investigation of differentially expressed genes (DEGs) under alkaline salt stress [18]. Further, other research has looked at how plants respond to alkali or high-pH stress. For example, the *OsLOL5* gene improves salt and alkali tolerance in transgenic Arabidopsis thaliana [19].

*Hosta plantaginea* is a perennial, rooted, herbaceous plant in the Asparagaceae family with high ornamental value that is commonly utilized in landscaping and environmental rehabilitation [20]. Some *Hosta* variants have features such as cold, shade, drought, and disease resistance [21]. In earlier investigations, *Hosta* ‘Guacamole’ reduced the negative impacts of drought stress by boosting the activity of antioxidant enzymes, including OEC and ascorbic acid [22]. *Hosta longipes* mitigated the effects of drought stress under ozone stress conditions by increasing its own antioxidant capacity and controlling hydrogen peroxide (H_2_O_2_) and superoxide radical (O_2_^−^) levels [23]. However, the geographic distribution of many *Hosta* species is limited by increased soil alkalinity in urban gardens. *Hosta* ‘Golden Cadet’, with yellow-green foliage, tightly spaced inflorescences, and some shade, cold, and salt tolerance, is a cultivar that has attracted attention for its distinctive foliage color and resistance to overwintering on open ground in the northeast [24]. The mechanism of salinity stress tolerance in *Hosta*, as well as the essential metabolic pathways and genes involved, are still unknown.

Salinity-induced osmotic stress produces fast plant damage, with leaf portions being particularly vulnerable [25,26]. Therefore, *H.* ‘Golden Cadet’ was chosen in this work to identify changes in several physiological markers and antioxidant enzyme activities in its leaf sections in response to NaHCO_3_ stress. Transcriptome sequencing was performed at time points where physiological features changed significantly, and key differentially expressed genes (DEGs) implicated in the response to alkaline stress were discovered. The current study lays the groundwork for elucidating *Hosta*’s physiological changes in response to alkaline stress conditions, as well as the molecular mechanism of its tolerance, with the goal of selecting and breeding salinity-tolerant *Hosta* germplasm resources. This study also serves as a reference for enriching the application of ornamental plants in the northeast region.

## 2. Results

### 2.1. Screening for Alkali Tolerance Thresholds in Hosta ‘Golden Cadet’

**Figure 1:**
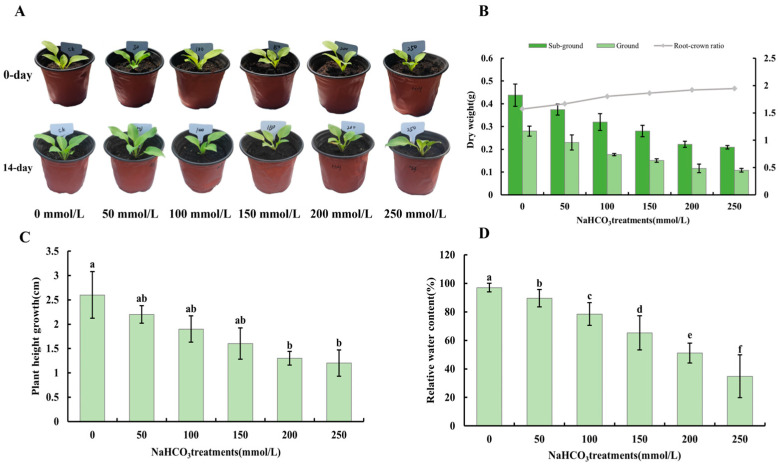
Alkali stress response of *H.* ‘Golden Cadet’: (**A**) *H.* ‘Golden Cadet’ before and after treatment with different concentrations of NaHCO_3_. (**B**) Above- and below-ground dry weights and root-crown ratios of *H.* ‘Golden Cadet’. (**C**) Changes in plant height of *H.* ‘Golden Cadet’. (**D**) Changes in relative water content of *H.* ‘Golden Cadet’ leaves. Note: letters (a–f) indicate statistically significant differences (*p* < 0.05).

*H.* ‘Golden Cadet’ exhibited substantial phenotypic variations after 14 d of treatment at various NaHCO_3_ concentrations (Figure 1A). Furthermore, when the stressor concentration increased, the aboveground and belowground dry weight, amount of plant height change, and relative water content all decreased (Figure 1B–D). At 200 mmol/L NaHCO_3_, the leaves’ relative water content ratio was 50%. Therefore, 200 mmol/L NaHCO_3_ was selected as the threshold alkali concentration for *H.* ‘Golden Cadet’ (Figure 1D).

### 2.2. Effect of NaHCO_3_ Stress on Osmoregulatory Substances and Antioxidant Activity of Hosta ‘Golden Cadet’

**Figure 2:**
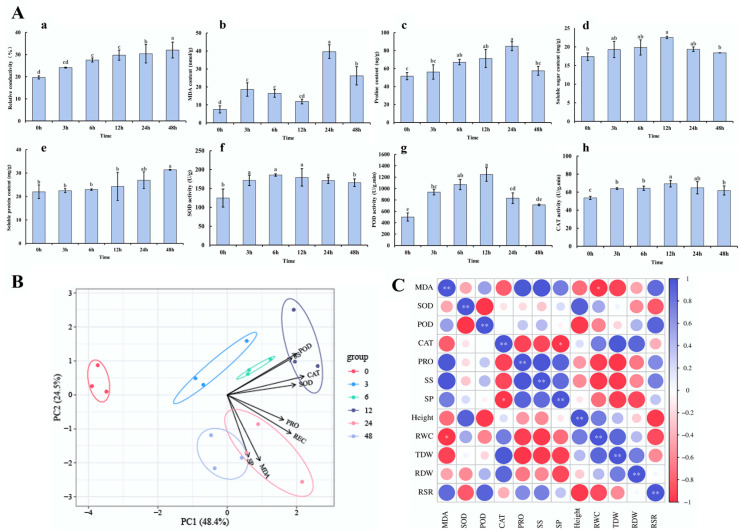
Changes in physiological indices of *H.* ‘Golden Cadet’ under alkali stress and principal component and correlation analysis: (**A**) Changes in physiological indices of leaf tissues at each time point. The results are summarized as follows: (**a**) REC; (**b**) MDA; (**c**) SOD; (**d**) POD; (**e**) CAT; (**f**) PRO; (**g**) SS; and (**h**) SP. The timeframe 0–48 h denotes the different times of exposure to alkali stress. (**B**) Principal component analysis of each physiological index. (**C**) Correlation analysis of plant growth indexes with 12 h physiological indexes. Notes: 0 h is before treatment. Error lines indicate mean ± SD (*n* = 3), and letters indicate statistically significant differences (*p* < 0.05), * and ** indicate *p* < 0.05 and *p* < 0.01, respectively.

To investigate the dynamic response of *H.* ‘Golden Cadet’ to NaHCO_3_ stress, *H.* ‘Golden Cadet’ seedlings were treated with 200 mmol/L NaHCO_3_ solution in the present study, and various physiological indexes related to the alkalinity tolerance of leaf tissues were determined at 0 h, 3 h, 6 h, 12 h, 24 h, and 48 h; then, various physiological indexes related to the alkali tolerance of their leaf tissues were measured (Figure 2A). The results show that the relative conductivity content of *H.* ‘Golden Cadet’ leaves rose linearly with time under NaHCO_3_ stress, reaching 1.63 times the control level after 48 h (Figure 2(Aa)). This indicates that *H.* ‘Golden Cadet’ had defective membrane plasma under alkali stress. The malondialdehyde (MDA) level in *H.* ‘Golden Cadet’ leaves gradually increased before peaking at 24 h (Figure 2(Ab)).

The analysis of osmoregulatory chemicals revealed that proline (PRO) content increased with the duration of alkali stress in *H.* ‘Golden Cadet’, with a trend of initial increase and then decrease under NaHCO_3_ stress until the final determination at 48 h (Figure 2(Ac)). The soluble sugar concentration under this stress treatment followed a similar pattern to that of PRO, peaking at 12 h (Figure 2(Ad)). During this procedure, soluble protein content gradually increased over time (Figure 2(Ae)). Thus, *H.* ‘Golden Cadet’ responded severely to NaHCO_3_ stress at 6 and 12 h.

Furthermore, the antioxidant activity assays showed that SOD and POD activity in *H.* ‘Golden Cadet’ followed an ‘increasing and decreasing’ pattern over time under NaHCO_3_ stress, with an increase in SOD activity within 6 h followed by a steady reduction up to 48 h (Figure 2(Af)). POD activity increased substantially for 12 h but then dropped significantly within 48 h (Figure 2(Ag)). In comparison, catalase (CAT) activity revealed a relatively moderate change under this stress condition, with a steady ascending and declining trend (Figure 2(Ah)). The activities of the above three enzymes in NaHCO_3_ stress-treated groups were always higher than the values recorded in the control seedlings, indicating that *Hosta* enhances its antioxidant capacity by increasing the activity of SOD and POD in the short term.

### 2.3. Principal Component and Correlation Analyses of the Response Pattern of Hosta ‘Golden Cadet’ to Alkali Stress

The principal component analysis of *H.* ‘Golden Cadet’ physiological indicators at each time point under NaHCO_3_ stress revealed that POD, CAT, and SS contributed the most to alkali stress response (Figure 2B). The correlation analysis of the growth indicators and physiological indicators at 12 h, the time point with the strongest response to alkali stress, revealed that RWC was positively correlated with REC and negatively correlated with MDA content (Figure 2C), indicating that *H.* ‘Golden Cadet’ responded to alkali stress via the antioxidant system and osmoregulation.

### 2.4. Transcriptome Sequencing, Assembly, and Functional Annotation Classification of Unigenes

To investigate the effects of NaHCO_3_ stress on *H.* ‘Golden Cadet’, we employed Illumina sequencing technology to sequence the RNA of *H.* ‘Golden Cadet’ at 0 h, 6 h, 12 h, and 48 h after alkali stress application. Leaf samples were sent for RNA sequencing. The samples were designated as (C, A6, A12, A48), with ‘C’ representing the control and ‘A’ indicating the *Hosta* variety (*H.* ‘Golden Cadet’), and for a total of 12 samples we obtained three biological replicates, resulting in the construction of 12 libraries. There were 277,601,484 raw readings in all. After quality filtering, 267,197,293 high-quality clean reads were recovered, totaling 80.17 gigabases (Gb), an average of 6.9 Gb per library. The sequencing quality was high, with Q20 and Q30 values of 98.71% and 96.26%, respectively, with GC content ranging from 45.21% to 48.83% for each library (Table S1). A total of 231,332 unigenes were obtained, with an N50 of 1286 bp, a minimum length of 301 bp, and a maximum length of 16,739 bp (Table S2). This demonstrates that the data obtained here are suitable for further analysis.

### 2.5. Differential Gene Expression Analysis

**Figure 3:**
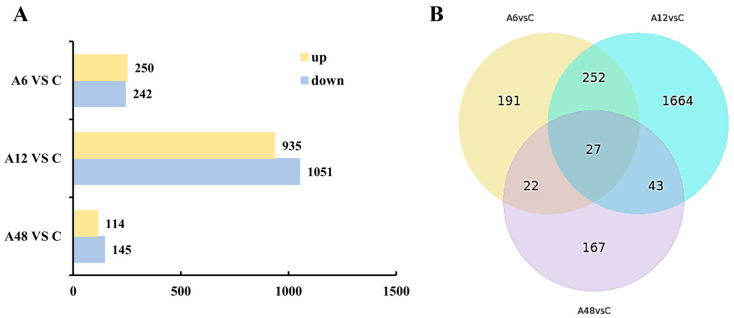
Functional annotation of DEGs in *H.* ‘Golden Cadet’ at different time points after NaHCO_3_ stress. (**A**) Statistics of up- and down-regulated DEGs in different controls. (**B**) Venn diagram of DEGs between different samples.

To assess the level of differential gene expression in each sample, three comparison groups were formed based on transcriptional differences at 6 h, 12 h, 48 h, and 0 h (control), resulting in 2366 DEGs. The differential gene expression results between NaHCO_3_ stress-treated genotypes and their controls revealed that there were 492 DEGs in A6 vs. C (250 up-regulated and 242 down-regulated genes), 1986 DEGs in A12 vs. C (935 up-regulated and 1051 down-regulated genes), and 259 DEGs in A48 vs. C (114 up-regulated and 145 down-regulated genes) (Figure 3). As can be seen, the number of DEGs increased from 0 h to 12 h and then dropped after 48 h of NaHCO_3_ administration. The greatest number of up-regulated and down-regulated DEGs appeared 12 h after stress treatment, indicating that the transcriptional expression pattern changes fast in the early stages of NaHCO_3_ stress. Many DEGs were expressed simultaneously at 6 h, 12 h, and 48 h, indicating that they continued to respond to alkali stress. Furthermore, the quantity of DEGs detected simultaneously correlates with the pattern of physiological markers.

### 2.6. Functional Annotation and Enrichment Analysis of DEGs

The top ten significantly enriched GO terms in Biological Processes (BPs), Cellular Components (CCs), and Molecular Functions (MFs) were selected based on the *p*-value associated with the number of DEGs for each term in each of the three comparison groups (A6 vs. C, A12 vs. C, and A48 vs. C) (Figure S1). It can be seen that *H.* ‘Golden Cadet’ is responsive to alkali stress through complex molecular regulation and physiological coordination between its intra- and extracellular environments due to the pathways involved in cellular activities and physiological responses early in the course of NaHCO_3_ stress; further, the genetic program of *Hosta* hairpins presents temporal differences in the signaling in response to environmental stimuli, and biotransport processes are also induced by prolonging the processing time.

**Figure 4:**
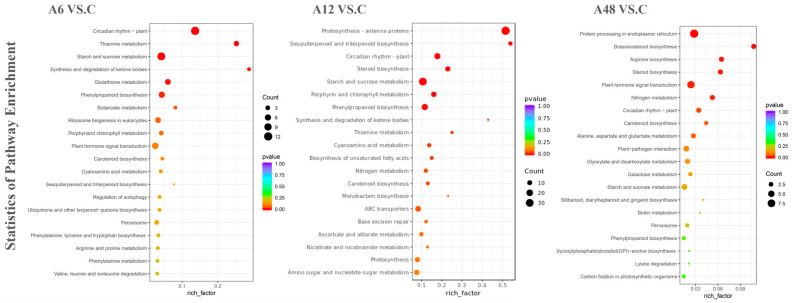
Top-20 KEGG enrichment pathways in *H.* ‘Golden Cadet’ exposed to salt stress for different time periods.

In addition, DEGs from the three comparison groups were evaluated for KEGG enrichment, with 20 enrichment pathways being labeled for each comparison group in order of *p*-value (Figure 4). Among these, more significantly enriched pathways were discovered at 12 h of therapy compared with 6 h and 48 h, which could be attributed to the diverse responses to NaHCO_3_ stress at different times. There were also a number of pathways that were significantly enriched at different processing times, including ‘Starch and sucrose metabolism’ (ko00500), ‘Plant hormone signal transduction’ (ko04075), ‘Circadian rhythm-plant’ (ko04712), ‘Phenylpropanoid biosynthesis’ (ko00940), ‘Steroid biosynthesis’ (ko00100), ‘Carotenoid biosynthesis’ (ko00906), ‘Sesquiterpenoid and triterpenoid biosynthesis’ (ko00909), ‘Thiamine metabolism’ (ko00730), ‘Amino sugar and nucleotide sugar metabolism’ (ko00520), and ‘Peroxisome’ (ko04146) (Figure 4). Among these, ‘Starch and sucrose metabolism’ was the most enriched KEGG pathway in 0–48 h, with 11, 30, and four DEGs being enriched at 6 h, 12 h, and 48 h, respectively, indicating that it is a significant metabolic route in *Hosta* plants in response to alkali stress. The KEGG pathways ‘Circadian rhythm-plant’, ‘Phenylpropanoid biosynthesis’, and ‘Carotenoid biosynthesis’ were co-enriched in the 0–48 h phase (Figure 4). Further, the DEG enrichment results for the three comparison groups revealed changes in pathway types and gene counts, showing that genes involved in plant metabolic pathways were activated under high-pH conditions.

### 2.7. Potential Transcription Factors of Hosta ‘Golden Cadet ’in Response to NaHCO_3_ Stress

**Figure 5:**
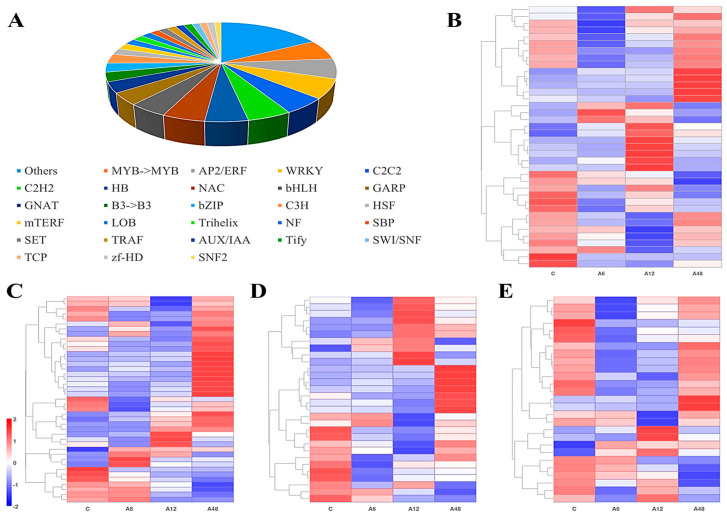
Changes in the expression levels of DEGs encoding transcription factors. (**A**) Statistical analysis of transcription factor families. Heatmap of DEGs involved in (**B**) MYB; (**C**) AP2/ERF; (**D**) MRKY; (**E**) C2C2 transcription factor family.

The transcriptome of *H.* ‘Golden Cadet’ leaves was examined for possible NaHCO_3_ stress-regulated transcription factors by using sequence comparisons based on TF families predicted from the Plant TFDB Arabidopsis database [27]. The analysis found that 634 DEGs encoded 50 TF families. The distribution is shown in Figure 5, including the MYB (7%), AP2/ERF (6%), WRKY (6%), C2C2 (5%), C2HC (5%), and HB (5%) families, which are significantly represented. In addition, transcription factor families that are not listed in the figure or Table S3 (with fewer than 1% of transcription factors) are classified as other. Figure 5B–E indicate that subsequent investigation of the first four TF families (AP2/ERF, MYB, WRKY, and C2C2) revealed that all genes were strongly up-regulated at different stress levels.

### 2.8. Validation of RNA-Seq Data with qRT-PCR

To confirm the correctness of the RNA-Seq results, we randomly selected 10 genes for qRT-PCR investigation. The results revealed that the relative expression of these candidate genes displayed a similar pattern to that discovered with RNA-seq (Figure S2).

### 2.9. DEGs Associated with Plant Hormone Signal Transduction

**Figure 6:**
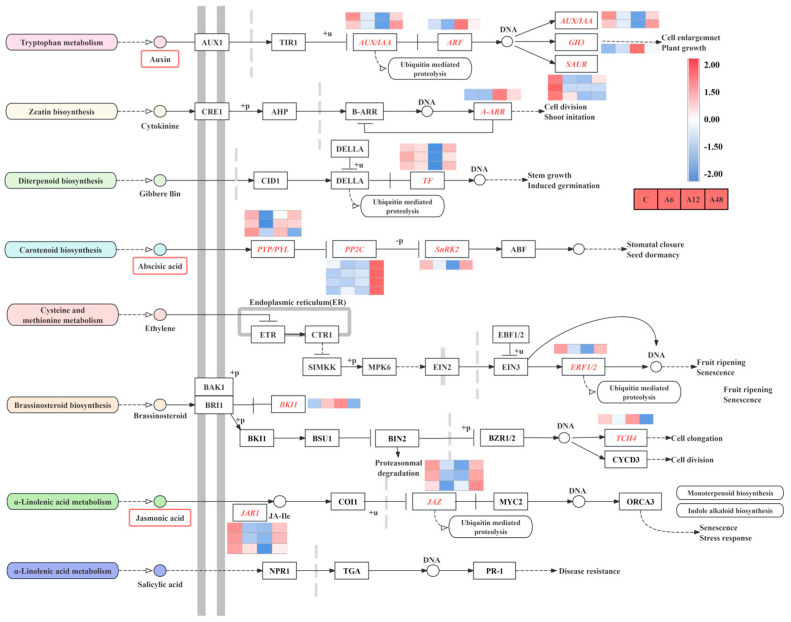
Analysis of the plant hormone signal transduction pathway. The color scale from Min (blue) to Max (red) refers to the expression value from low to high. Uppercase letters indicate genes that encode enzymes. Solid arrows represent established biosynthesis steps and broken arrows indicate the involvement of multiple enzymatic reactions.

In this study, KEGG enrichment analysis found that the phytohormone signaling pathway was a significant enrichment route. A total of 28 DEGs were discovered in different stages of alkali stress therapy (Figure 6). The study of DEGs related to hormone signaling revealed that alkali stress induces numerous hormone signaling pathways. Among these, the IAA, ABA, and JA signaling pathways were considerably enriched, activating a large number of genes with differential expression. In the IAA signaling pathway, *GH3* (Cluster-32038.43493) and growth hormone response factor *ARF* (Cluster-32038.37896) were up-regulated, *SAUR*-related genes were down-regulated, and several ABA signaling pathway components, including *PYL*, *PP2C*, and *SnRK2*, were differentially regulated. *JAR1_4_6* and *JAZ* were down-regulated in the JA signaling pathway across all stress periods.

### 2.10. Starch and Sucrose Metabolism Pathways in Response to Alkali Stress

**Figure 7:**
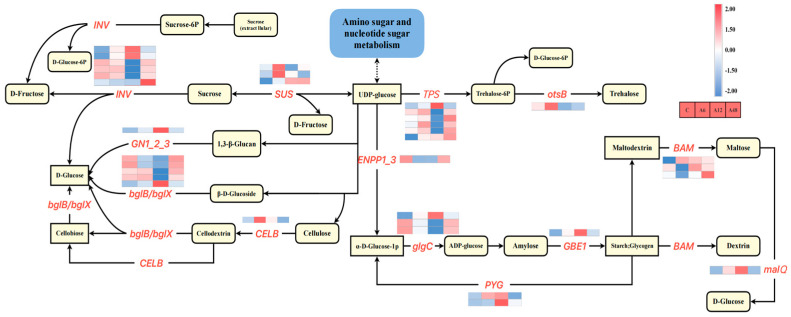
Analysis of starch and sucrose metabolic pathways. The color scale from Min (blue) to Max (red) refers to the expression value from low to high. Uppercase letters indicate genes that encode enzymes. Solid arrows represent established biosynthesis steps.

In this study, a statistical analysis of the DEGs enriched within the pathways of starch and sucrose metabolism (Figure 7) revealed that 38 DEGs were obtained in the three comparison groups after deleting duplicate treatments for DEGs enriched in groups A6 vs. C, A12 vs. C, and A48 vs. C. Genes encoding β-glucosidases for starch and sucrose metabolism (*bglX* and *bglB*) were the most abundant across all stress periods, and their expression was mostly down-regulated. Three β-amylase (*BAM*) genes showed variable expression, with two being up-regulated (Cluster-32038.38748 and Cluster-32038.41979) and one down-regulated (Cluster-32038.39185). Furthermore, many distinct genes encoding glucose-1-phosphate adenylyltransferase, trehalose 6-phosphate synthase/phosphatase, and beta-fructofuranosidase were expressed at higher levels after 12 h of alkali stress treatment compared with the other two occasions.

### 2.11. DEGs Associated with the Phenylpropanoid Biosynthesis Pathway

**Figure 8:**
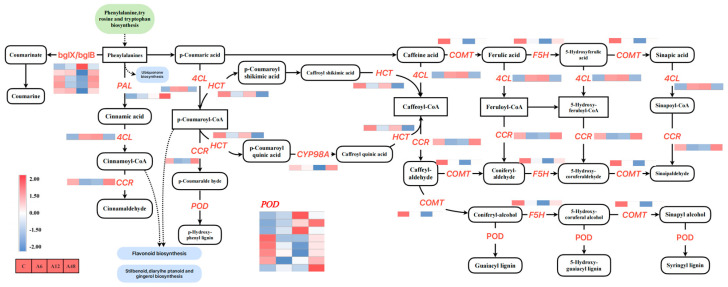
Analysis of phenylpropanoid biosynthetic pathways. The color scale from Min (blue) to Max (red) refers to the expression value from low to high. Uppercase letters indicate genes that encode enzymes. Solid arrows represent established biosynthesis steps.

A total of 21 DEGs encoding nine enzymes in this pathway were tested at various stress levels. During alkali stress, the expression of most genes producing β-glucosidase (*bglX* and *bglB*), *CCR*, *CYP84A*, and *CYP98A* decreased (Figure 8). In contrast, *PAL* (Cluster-32038.52754), *4CL* (Cluster-32038.45477), and *HCT* (Cluster-32038.38060) were up-regulated for expression during stress. Furthermore, as alkali stress progressed, the expression of peroxidase genes increased.

## 3. Discussion

### 3.1. Hosta ‘Golden Cadet’ Seedling Leaves Respond to Alkali Stress by Increasing Osmoregulatory and Antioxidant Capacity

Salt and alkali stress have a negative impact on plant growth and development; in the present research study, *H.* ‘Golden Cadet’ experienced growth inhibition as a result of alkali stress. Plants’ physiological responses to saline and alkaline stress contribute to the buildup of reactive oxygen species (ROS) in cells [28]. Low amounts of ROS can activate signaling pathways, whereas high levels of ROS can degrade cell membrane integrity and promote membrane lipid peroxidation, affecting relative conductivity [29,30,31]. Thus, variations in relative conductivity indicate the degree of cell membrane damage as a result of osmotic stress [32]. The salt-tolerant REC of *Glaux maritima* leaf increased with the increase in salt concentration [33]. This is consistent with the findings of the current investigation, which indicate that under alkali stress, *H.* ‘Golden Cadet’ chloroplasts maintain intracellular homeostasis by accumulating very large amounts of ions to protect the membrane. While malondialdehyde (MDA) is the primary result of polyunsaturated fatty acid peroxidation, increasing its levels exacerbates membrane damage [34]. In this study, MDA content exhibited no significant trend from 0 to 12 h of stress treatment, but it increased significantly at 24 h. The MDA content in *H.* ‘Golden Cadet’ increased considerably between 0 and 12 h of stress treatment, and it increased substantially more at 24 h. This could be due to increased formation of ROS in *H.* ‘Golden Cadet’ after 24 h of alkali stress treatment, resulting in high MDA concentration.

Plants also respond to unfavorable conditions by storing extra proline, soluble sugars, and other osmoregulatory chemicals that lower cellular osmotic potential. Both can also be employed to protect cytoplasmic enzymes and membranes, increasing buffering against damage caused by salinity, dehydration, and other types of abiotic stress [35]. In the current work, *H.* ‘Golden Cadet’ was exposed to NaHCO_3_ stress, which disrupted its physiological and metabolic functions, and it responded by accumulating proline. Protein synthesis can also be used as a key stress signal in plants in response to salt stress, and this response varies based on the plant species, developmental stage, and duration and intensity of exposure [36]. For example, salt stress raises protein levels in *Zoysia macrostachya* [37]. Under both salt and alkaline stress, the soluble protein level in *Xanthoceras sorbifolia* [38] exhibited an ‘increasing and decreasing’ trend. This is congruent with the current work, which showed that *H.* ‘Golden Cadet’ increased its soluble sugar and soluble protein concentrations after 12 h of alkali stress treatment, indicating that it responds to stress in the short term via osmoregulation.

Plants increase antioxidant enzyme activity to remove excess ROS and to maintain the plant’s internal dynamic balance under stress, preventing cell membrane damage. SOD, POD, and CAT are important protective enzymes that help eliminate ROS and promote tolerance to abiotic stressors. The induction of antioxidant systems is determined by the severity of the stress the plant is subject to [39]. In the present study, we found that alkali stress dramatically increased SOD content, which could imply that harmful superoxide caused plant damage under alkali stress. POD and CAT activities, on the other hand, exhibited an initial increase before decreasing. It appears that *H.* ‘Golden Cadet’ can respond to alkali stress in the short term by boosting the activity of antitrophic enzymes.

### 3.2. Hosta ‘Golden Cadet’ Leaves Respond to Alkali Stress by Activating Transcription Factors

According to RNA-seq analysis, the number of DEGs peaked after 12 h of alkali stress treatment and gradually declined, which was compatible with the trend of the observed physiological markers and also consistent with findings on Arabidopsis performance under salt stress [40]. This shows that genes can respond quickly to alkali stress and gradually adjust to the stress environment. In addition, as regulatory components in the transcriptional network, TFs are engaged in a variety of activities such as plant development, hormone signaling, and stress response, and are frequently seen as the most essential regulators of multiple target gene expression [41,42]. In this study, most of the identified TFs belonged to the AP2/ERF, MYB, WRKY, bHLH, and NAC families, which are involved in plant response to abiotic stress [43]. For example, the MYB, WRKY, NAC, AP2/EREBP, bHLH, and bZIP transcription factor families are activated in response to salinity stress in *Medicago sativa* L. [44]. Eleven genes associated with bHLH, MYB, and WRKY TFs were detected in the leaf transcriptome of *Prunus persica* L. and *P. amygdalus x P. persica* under salinity stress [45]. The WRKY gene family plays a crucial role in biological processes and abiotic stress responses, including ABA sensitivity and plant nutrition control [46,47]. Salt-tolerant *Zea mays* L. inbred lines (L87) exposed to salt stress exhibited significant differences in the expression of WRKY transcription factors [48]. AP2/ERF, the biggest family implicated in plant salt stress response, has the potential to regulate plant growth and development [49,50]. The overexpression of the AP2/ERF transcription factor *OsERF19* can improve *Oryza sativa* L.’s tolerance to salt stress [51]. In addition, MYB members play important roles in the regulation of plant secondary metabolism, cellular morphological changes, and stress responses [52,53]. When *Triticum aestivum* L. is exposed to salt stress, it responds by activating the MYB transcription factors *MYB3*, *MYB4*, *MYB13*, and *MYB59* [54]. In this study, we found that numerous AP2/ERF, WRKY, and MYB transcription factors were differentially expressed in different stages of alkali stress, implying that *Hosta* activates them in response to alkali stress treatment. As a result, these transcription factors play an active part in *Hosta*’s alkali tolerance, and their functions require further investigation.

### 3.3. Hosta ‘Golden Cadet’ Responds to Alkali Stress by Activating Phytohormone Signaling

Phytohormone signaling acts as a central regulator of alterations in salt-responsive gene expression [55]. In this study, GO and KEGG enrichment revealed that the phytohormone signaling pathway was highly enriched in *H.* ‘Golden Cadet’ under alkali stress. The GH3 family and growth hormone response factor *ARF* genes were up-regulated in the growth hormone signaling pathway, while the SAUR family genes were down-regulated, implying that IAA responds to alkali stress by regulating the up-regulation or down-regulation of various DEGs, as well as helping to counteract the toxic effects exerted by stress [56]. In the ABA signaling pathway, ABA-dependent transcription factors *ABF*, *MYC*, and *MYB* bind to and stimulate the activation of particular areas on the promoters of salt stress-responsive genes [57]. In *Lilium pumilum*, ABA signaling genes are dramatically altered in response to salt stress [24]. While the first step of ABA signaling involves *PYR*/*PYL*/*RCAR*-encoded proteins and phosphatases/kinases that have antagonistic functions against the proteins encoded by *PP2C* and *SnRK2*s, respectively, ABA signaling allows transcription factors to be expressed by genes that are regulated by *SnRK2* [58,59]. In this study, *H.* ‘Golden Cadet’ was subjected to alkali stress, and *PP2C* up-regulated the expression and inhibited the activity of *SnRK2*-encoded enzymes as a means to regulate the physiological and biochemical processes of the plant to alleviate leaf damage and enhance its ability to survive. This corresponds to the findings of a prior investigation [56,60]. Furthermore, earlier research has shown that the JA signaling pathway has a role in plant growth, development, and stress response [60]. JA induced the expression of a large number of downstream genes during the alkali stress response stage in *H.* ‘Golden Cadet’. Among them, *JAR1* and *JAZ* were down-regulated for expression. This suggests that when *H.* ‘Golden Cadet’ was exposed to alkali stress, the JA signaling pathway was activated, increasing the resistance of *H.* ‘Golden Cadet’ to alkali stress.

### 3.4. Hosta ‘Golden Cadet’ Responds to Alkali Stress by Increasing Starch and Sucrose Metabolizing Capacity

Starch and sucrose are two of the most common carbohydrates in plants; they play an important role in energy storage and supply; their content determines cellular osmotic pressure and leaf water content; and their metabolism is regulated by related degradative enzymes, such as amylase and gluconeogenic enzymes, and is thus essential to regulating plant responses to abiotic stress [61,62]. In this study, we found the starch and sucrose metabolic pathways were considerably enriched under alkali stress, with a total of 38 DEGs identified, and that the majority of the genes were differently expressed at 12 h, agreeing with changes in the relevant physiological markers. Four beta-amylase genes were enriched, with two being up-regulated and two being down-regulated. Drought and salt stress can increase sweet potato’s β-amylase activity and starch breakdown [63]. This outcome is consistent with our observations. Furthermore, plants reduce salinity stress by up-regulating the expression of enzyme genes such as converting sucrose synthase, trehalose 6-phosphate synthase, trehalose phosphate phosphatase, and starch synthase [64]. Alkali stress greatly increased the expression of genes producing β-glucosidase and alginate 6-phosphate synthase, whereas trehalose 6-phosphate synthase acts as a signaling and regulating agent of sucrose levels in plants, which can improve *Hosta* alkali tolerance. In *H.* ‘Golden Cadet’, three sucrose synthase genes (Cluster-32038.50028, Cluster-32038.44219, and Cluster-32038.25198) were differentially up-regulated, which can be used to reduce osmotic stress and increase energy supply by synthesizing soluble sugars. As shown above, starch hydrolysis, glucose release, and alginate synthesis resulted in the buildup of additional sucrose, glucose, and alginate in *Hosta*. These carbohydrates not only served as osmotic agents to keep the cells’ osmotic pressure constant, but they also provided nutrients and energy for *Hosta’s* growth and development, increasing its tolerance to salt stress.

### 3.5. Hosta ‘Golden Cadet’ Responds to Alkali Stress by Regulating Phenylpropane Biosynthesis

The phenylpropane biosynthetic pathway is a prominent mechanism for the biosynthesis of flavonoid molecules, and the compounds it produces are an important class of plant secondary metabolites found throughout plants [64]. This pathway’s involvement in alkali stress is to help plants minimize stress damage while also inducing the adaptability and survival of the plant body under stressful conditions by increasing lignin synthesis capacity [65]. *Sophora Alopecuroides* resistance to salt stress damage is enhanced by increasing its lignin concentration [66]. Arabidopsis thaliana enhanced lignin concentration by overexpressing the NAC transcription factor *AgNAC1*, which improves SOD and POD activity under drought and salt stress [67]. *H.* ‘Golden Cadet’ greatly enriched this route throughout the alkali stress cycle. This route is controlled by genes such as *4CL*, *CCR*, and *POD*. *4CL* is a critical gene that links lignin synthesis in the phenylpropane metabolic pathway, and it can be triggered when the plant is exposed to abiotic stress [68]. This study in *H.* ‘Golden Cadet’ demonstrated that *4CL* activity was triggered and expression was dramatically increased following alkaline stress. *CCR* is an important gene for lignin biosynthesis, which is mediated by stimulating the creation of five cinnamoyl-coenzyme A esters, which serve as substrates for the production of the corresponding cinnamaldehyde [68]. The findings of this investigation revealed that all of the genes governing *CCR* expression were down-regulated, implying that *H.* ‘Golden Cadet’ improves its resistance to alkali stress by increasing lignin synthesis. *POD* is an action enzyme in the final step of lignin production that participates in the lignin polymerization reaction, which increases the plant’s stress resistance [69]. In this study, the expression of genes regulating *POD* gradually shifted from being down-regulated to being up-regulated as the stress time increased, demonstrating that *Hosta* responds to alkali stress by boosting peroxidase activity in the synthesis pathway to reduce the effects of stress.

Thus, in order to achieve better growth in urban alkaline soil, *H.* ‘Golden Cadet’ would need to be cultivated in sparse woodland with a cultivation density of 30 cm × 40 cm [70,71]. This may increase its starch and sucrose metabolism and regulate phenylpropane biosynthesis, osmotic management, and antioxidant capacity by enhancing its photosynthetic yield, all of which help to mitigate the harmful consequences of alkali stress. If the cultivation environment has been exacerbated by alkalization, the alkali tolerance of *H.* ‘Golden Cadet’ can be improved with the topical administration of plant growth regulators such as IAA, ABA, and JA [56,60].

## 4. Materials and Methods

### 4.1. Experimental Materials and Treatment

The test material (*H.* ‘Golden Cadet’) used in this study was a *Hosta* cultivar that can overwinter and thrive in open ground in the northeast. The test included healthy tissue culture seedlings grown under similar conditions. *H.* ‘Golden Cadet’ seedlings in culture flasks were transplanted into plastic pots with a diameter of 100 mm and a height of 88 mm, filled with a mixture of charcoal soil, perlite, and sand. Seedlings were housed indoors at a temperature of 25 °C and humidity of 70–80% and were weighed and hydrated daily. After 30 d of incubation and growth, the plants were irrigated with NaHCO_3_ solutions at 0 (distilled water), 50, 100, 150, 200, and 250 mmol/L. The relevant growth parameters were measured on the 14th d after treatment, and the ratio of fresh weight of leaves in the treated groups to that of untreated leaves was used to calculate the ratio of fresh weight of leaves, or leaf relative water content. The NaHCO_3_ concentration that lowered the leaf relative water content to 50% was chosen as the alkali threshold concentration for *H.* ‘Golden Cadet’ [24]. Based on the results of the preceding tests, the plants were treated with 200 mmol/L NaHCO_3_ solution as a stress condition for 3 h, 6 h, 12 h, 24 h, and 48 h, with pre-treatment serving as the control (0 h). Afterwards, the plant leaves were gathered, frozen in liquid nitrogen, and kept at −80 °C for RNA sequencing and the evaluation of physiological indicators.

### 4.2. Physiological Trait Determination

The relative conductivity of *H.* ‘Golden Cadet’ leaves was measured by obtaining 0.2 g of fresh leaf samples, washing them three times with deionized water, and immersing them in sealed tubes containing 20 mL of deionized water at 25 °C for 5 h. The conductivity (S1) of the leaf solution was measured by using a conductivity meter (DDS-307; Shanghai, China). The sealed tubes containing the solutions were then immersed in boiling water at 100 °C for 20 min to determine conductivity (S2) following the release of all electrolytes. Leaf electrolyte leakage was evaluated by using the method developed by Dionisio-Sese and Tobita [72] and computed as follows:

REC = (S1/S2) × 100%,(1)

Next, 0.1 g samples to be examined for additional physiological indices were weighed in centrifuge tubes, crushed by using liquid nitrogen, and analyzed by using assay kits. The kits (catalog numbers AKAM 003C, AKPL 008C, AKPR 001C, and AKFA 013C, respectively; Boxbio, Beijing, China) were used to measure the levels of proline, soluble sugars, soluble proteins, and MDA, respectively. SOD, POD, and CAT were among the three antioxidants whose enzymatic activities were also assessed (catalog numbers AKAO 001C, AKAO 005C, and AKAO 003-2C, respectively; Boxbio, Beijing, China). According to the guidelines, a UV/visible spectrophotometer (UV1050) was used to test the aforementioned physiological parameters by utilizing particular operating techniques and calculation algorithms. In order to increase the study’s dependability, three replication samples were made for each treatment sample in this experiment.

### 4.3. RNA Extraction, Library Construction, and Sequencing

*H.* ‘Golden Cadet’ leaf samples treated with NaHCO_3_ for 0 h, 6 h, 12 h, and 48 h were named 0 h control (C-1, C-2, and C-3), 6 h treatment (A6-1, A6-2, and A6-3), 12 h treatment (A12-1, A12-2, and A12-3), 12 h treatment (A12-1, A12-2, and A12-3), and 48 h treatment (A48-1, A48-2, and A48-3) groups. Total RNA was isolated from 12 samples using TRIzol reagent (Invitrogen, Carlsbad, CA, USA) as per the manufacturer’s instructions. An Agilent 2100 bioanalyzer (Agilent Technologies, Santa Clara, CA, USA) was utilized to correctly detect RNA integrity and total amount, and library construction was carried out. After library building was completed, initial quantification was performed by using a Qubit 2.0 fluorometer (Gentier 96E, Suzhou Tianlong Biotechnology Co., Ltd., Suzhou, China), followed by insert size detection with the Agilent 2100 bioanalyzer. Novozymes Biotechnology Ltd. (Beijing, China) sequenced all libraries and generated paired-end reads by using the Illumina Novaseq^TM^ 6000 platform (LC Sciences, Houston, TX, USA). The raw sequence reads have been submitted to NCBI’s Short Read Archive (SRA) under the accession number PRJNA1187356.

### 4.4. Analyses of Sequencing Data and Functional Annotation

Clean reads were obtained by eliminating reads with splice and ploy-N sequences, as well as low-quality reads, from the raw data by using FastQC software (Cock PJ et al., 2010, http://www.bioinformatics.babraham.ac.uk/projects/fastqc/, accessed on 25 July 2024). Trinity software 2.6.6 [73] was used to create transcripts from high-quality readings. The expression levels of all constructed unigenes were measured and normalized to FPKM (fragments per kb per million reads). The gene function was established using the following databases: NCBI Non-Redundant (Nr) Proteins, Protein Families (Pfam), Swiss-Prot, Gene Ontology (GO), COG/KOG, and KEGG. The transcription factors (TFs) were then predicted by using the Plant TFDB database (http://planttfdb.gao-lab.org/index.php, accessed on 5 August 2024).

### 4.5. Differential Expression and Functional Enrichment Analysis

The DEGs were analyzed by using Gene Ontology (GO) and the Kyoto Encyclopedia of Genes and Genomes (KEGG) to determine unigene expression levels by calculating TPM (transcripts per kilobase per million mapped reads of the exon model). The R package edgeR [74] was used with the criterion log2 (fold change) > 1 or <−1 to identify differentially expressed unigenes, with a statistical significance *p*-value of <0.05. GO Functional Enrichment Analysis: The number of genes per phrase was ascertained after all significant DEGs were allocated to each term in the GO database. Significantly enriched GO keywords in the DEGs were then found by using the hypergeometric test. The *p*-value indicates the significance of the GO word enrichment analysis. Similarly, to identify the significantly enriched pathways of the DEGs with the corresponding *p*-values, KEGG significant enrichment analysis and hypergeometric tests were used.

### 4.6. Quantitative Real-Time PCR

To ensure the validity of the transcriptome sequencing results, 10 DEGs were randomly chosen from the transcriptome data and subjected to RT-qPCR. RNA was extracted from the leaves by using the RNA Easy Fast Plant Tissue Kit (TianGen, Beijing, China). The Takara Reverse Transcription Kit (TaKaRa, Beijing, China) was used to reverse-transcribe the whole RNA that was collected. The SYBR Green Mix kit (TaKaRa, Beijing, China), a Gel Doc Go imaging system, and a quick real-time detection system were then used for qRT-PCR. HI-actin was chosen as the internal reference gene, and qRT-PCR primers were created by using the NCBI Primer-Blast program based on the encoded gene sequences (Table S4). The expression levels of target genes under various treatments were calculated and calibrated by using the relative quantitative approach 2^(−ΔΔCT)^.

### 4.7. Statistical Analysis

The experimental data were processed and analyzed by using Microsoft Excel 2021 and SPSS 27.0 software. One-way ANOVA, principal component analysis (PCA), and Pearson correlation analysis were used for comparison across groups. The results were expressed as means ± standard deviations. The main component and correlation analyses for graphing were carried out by using R 4.3.1 software. Statistical significance was defined as *p* < 0.05, with superscript letters indicating significant differences across groups.

## 5. Conclusions

In this work, alkali stress was used to treat *Hosta* ‘Golden Cadet’. As the length of alkali stress treatment increased, the membrane plasma of *Hosta* ‘Golden Cadet’ was damaged, and its osmoregulatory ability was improved by increasing osmoregulatory chemicals such as proline and soluble carbohydrates. It also improved its antioxidant ability by enhancing superoxide dismutase and peroxidase activity. In addition, *Hosta* ’Golden Cadet’ responded to alkali stress by activating transcription factor families such as MYB, AP2/ERF, and WRKY, as well as genes involved in the synthesis of growth hormone, abscisic acid, and jasmonic acid in phytohormone signaling. *Hosta* ‘Golden Cadet’ has improved osmoregulatory and antioxidant properties via enhancements of starch and sucrose metabolism and control of phenylpropane production to reduce the deleterious effects of alkali stress. This study will aid in the investigation of the molecular mechanisms of *Hosta* ‘Golden Cadet’ in response to alkali stress, as well as the development of a metabolic profile of its reactions. The goal is to improve our understanding of how *Hosta* plants adapt to harsh environmental conditions, as well as to provide fresh ideas for research into boosting saline and alkali tolerance in *Hosta* species. This can further aid in the use of decorative plants in saline soil management.

## Data Availability

The data have been deposited to the National Center for Biotechnology Information (NCBI) under accession number PRJNA1187356.

## Supplementary Materials

The following supporting information can be downloaded at: https://www.mdpi.com/article/10.3390/plants14040593/s1, Table S1: Statistical analysis of the raw and clean sequencing of the RNA-seq library; Table S2: Distribution of spliced transcript lengths; Table S3: Differentially expressed TFs from the common DEGs; Table S4: Primers of the qRT-PCR verification test. Figure S1: Top-10 GO enrichment terms in *H.* ‘Golden Cadet’ exposed to salt stress for different time periods. BP, CC, and MF are short for biological process, cellular component, and molecular function, respectively. Figure S2: qRT-PCR validation of the expression levels of ten DEGs identified by RNA-seq. The left *Y*-axis indicates the relative gene expression levels analyzed by qRT-PCR, and the right *Y*-axis represents the FPKM values obtained by RNA-seq. The *X*-axis represents different samples.

## Abbreviations

The following abbreviations are used in this manuscript:

*AUX*/*IAA*	Auxin-responsive protein IAA
*ARF*	Auxin response factor
*GH3*	Auxin responsive GH3 gene family
*SAUR*	SAUR family protein
*ARR-A*	Two-component response regulator ARR-A family
*PLF4*	Phytochrome-interacting factor 4
*PYL*	Abscisic acid receptor PYR/PYL family
*PP2C*	Protein phosphatase 2C
*SnRK2*	Serine/Threonine-protein kinase SRK2
*ERF1*	Ethylene-responsive transcription factor 1
*BKI1*	BRI1 kinase inhibitor 1
*TCH4*	Xyloglucan:xyloglucosyl transferase TCH4
*JAR1*	Jasmonic acid-amino synthetase
*JAZ*	Jasmonate ZIM domain-containing protein
*SUS*	Sucrose synthase gene
*bglB**/bglX*	beta-glucosidase gene
*ENPP1_3*	Endoglucanase gene
*TPS*	Trehalose 6-phosphate synthase/phosphatase gene
*PYG*	Glycogen phosphorylase gene
*malQ*	4-alpha-glucanotransferase gene
*GBE1*	1,4-alpha-glucan branching enzyme gene
*BAM*	beta-amylase gene
*INV*	beta-fructofuranosidase gene
*GN1_2_3*	Glucan endo-1,3-beta-glucosidase 1/2/3
*glgC*	Glucose-1-phosphate adenylyltransferase
*otsB*	Trehalose 6-phosphate phosphatase
*PAL*	Phenylalanine ammonia-lyase gene
*4CL*	4-coumarate--CoA ligase gene
*CCR*	Cinnamoyl-CoA reductase gene
*POD*	Peroxidase gene
*HCT*	Shikimate O-hydroxycinnamoyltransferase gene
*CYP98A*	5-O-(4-coumaroyl)-D-quinate 3′-monooxygenase gene
*COMT*	Caffeic acid 3-O-methyltransferase/Acetylserotonin O-methyltransferase gene
*CYP84A*	Ferulate-5-hydroxylase gene
