# Supplementary figures and images for: Combining Physiology and Transcriptome to Reveal Mechanisms of Hosta ‘Golden Cadet’ in Response to Alkali Stress
Source: Plants (Basel). 2025 Feb 15;14(4):593. doi: 10.3390/plants14040593 (PMC11858970; doi:10.3390/plants14040593)

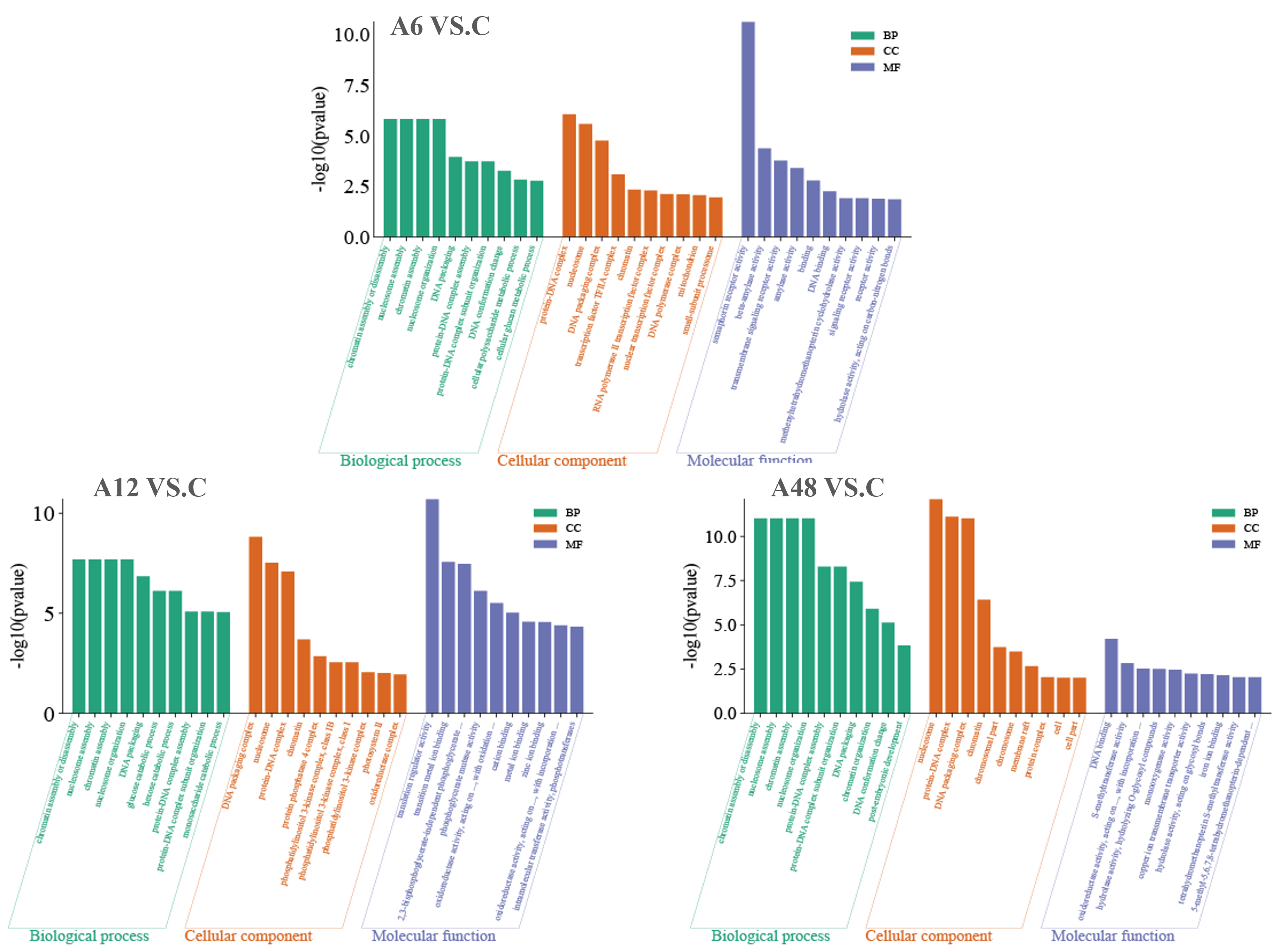

Supplement: Supplementary file 1 [file plants-14-00593-s001.zip › Figure S1.png]

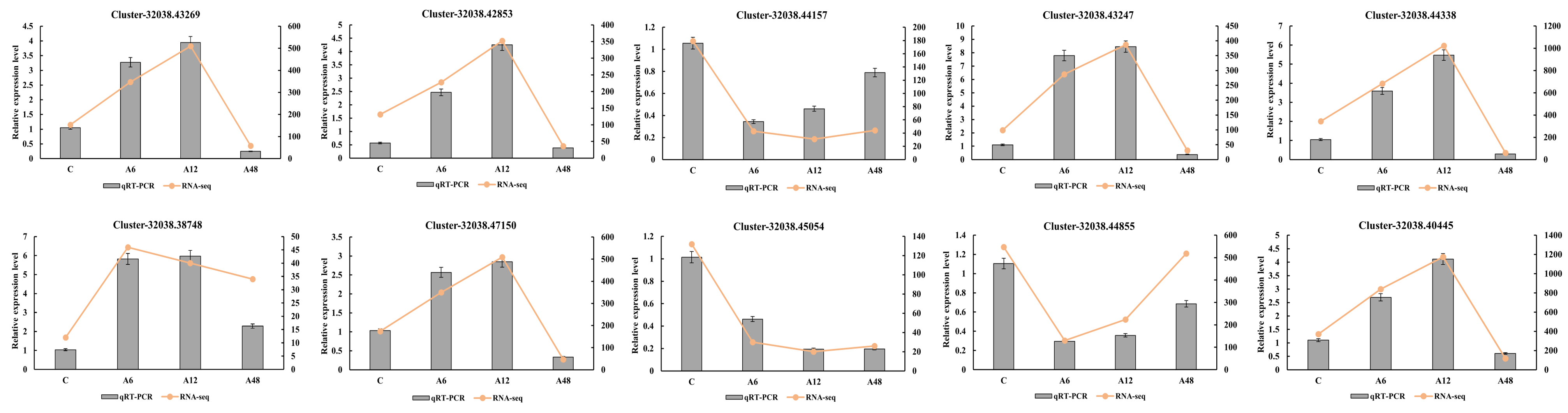

Supplement: Supplementary file 1 [file plants-14-00593-s001.zip › Figure S2.png]
